# Neurophysiological Analysis of Intermanual Transfer in Motor Learning

**DOI:** 10.3389/fnhum.2019.00135

**Published:** 2019-04-18

**Authors:** Ryuji Oosawa, Risa Iwasaki, Tomotaka Suzuki, Shigeo Tanabe, Kenichi Sugawara

**Affiliations:** ^1^Department of Rehabilitation, Shinshu University Hospital, Matsumoto, Japan; ^2^Saiseikai Kanagawa Hospital, Kanagawa-ku, Japan; ^3^Division of Physical Therapy, Faculty of Health and Social Work, Kanagawa University of Human Services, Yokosuka, Japan; ^4^Faculty of Rehabilitation, School of Health Sciences, Fujita Health University, Toyoake, Japan

**Keywords:** intermanual transfer, motor control, motor learning, motor evoked potential, transcranial magnetic stimulation

## Abstract

The purpose of this study was to examine the effect of motor training on motor imagery (MI), by comparing motor performance and motor cortex excitability changes with and without intermanual transfer of motor learning. Intermanual transfer was investigated in terms of excitability changes in the motor cortex and motor performance from right hand training to left hand performance. Participants were assigned to a transfer training group and a control group. We recorded motor evoked potentials (MEPs) induced by transcranial magnetic stimulation (TMS), applied to the left extensor carpi radialis (ECR) both with and without intermanual transfer. The results showed that after learning by the right hand, MEPs decreased during left hand MI. MEPs during MI were significantly decreased by unilateral training in the transfer training group. Since intermanual transfer plays an important role in stabilizing performance by the contralateral side, this result suggests that unilateral training decreases MEPs during MI on the contralateral side. In the control group, without right hand training, MEPs significantly increased after left hand training during MI. In the trained side, we found increased excitability in the agonist muscle area of the primary motor cortex. However, in the untrained side, excitability decreased in the homonymous muscle area of the primary motor cortex. This constitutes an increase in inhibitory effects and suggests that excitability changes in the respective neural circuit contribute to skilled performance by the ipsilateral and contralateral sides in the same motor task.

## Introduction

Intermanual transfer is the phenomenon in which unilateral training induces improvements in contralateral motor performance (Ruddy and Carson, 2013). It has often been reported that unilateral strength training increases the strength of the contralateral limb (Munn et al., 2004; Carroll et al., 2006; Dragert and Zehr, 2013). It was also confirmed that unilateral improvements in the accuracy of motor control are translated to motor performance improvements in the contralateral limb (Laszlo et al., 1970; Imamizu and Shimojo, 1995).

During rehabilitation therapy, exercise using the non-paralytic limb is sometimes applied in stroke patients and patients with motor disorders to improve contralateral performance. Magnus et al. (2010) conducted a study by applying cross-training for 4 weeks, after unilateral limb immobilization, using a shoulder sling and swathe, to investigate the effects on muscle strength, muscle size, and muscle activation. The study showed that strength training of the non-immobilized limb benefited the immobilized limb in terms of muscle size and strength. Ausenda and Carnovali (2011) examined the ability of intermanual transfer in facilitating the motor skills of a paretic hand in stroke patients. Patients were asked to execute the nine-hole peg test using the non-paretic hand, 10 times per day for three consecutive days. The results suggested that this regime improved the ability of the affected hand. Taken together, these investigations suggest that intermanual transfer could be a useful approach for rehabilitation of patients with motor disorders.

Recently, new developments on motor imaging research have clarified the neurophysiological mechanisms of intermanual transfer. Notably, a study using transcranial magnetic stimulation (TMS) showed that improvement in motor performance of the contralateral hand, induced by unilateral exercise, is associated with excitability of the contralateral motor cortex (Camus et al., 2009). Moreover, a study using functional magnetic resonance imaging found that unilateral movement not only increases the activity of the ipsilateral motor cortex, but that the ipsilateral sensorimotor cortex, premotor areas, and contralateral cerebellum are also activated (Dai et al., 2001; van Duinen et al., 2008). Thus, the mechanism of intermanual transfer may involve complementary changes in excitability between the right and left hemispheres.

However, there is no information about how spatial and other factors affect motor cortex excitability effects in the transfer of motor skills from the trained to the untrained side. In previous studies, most research designs involved comparing motor performance on the untrained side before and after exercises performed using the trained side (Carroll et al., 2008; Camus et al., 2009; Dickins et al., 2015). As a result, the effects on the untrained side may have been induced by sensory feedback caused by muscle contraction. Therefore, it is unlikely that changes in the untrained side were entirely induced by changes in the trained side.

To avoid this problem, we used motor imagery (MI) involving the untrained side. MI may be defined as a dynamic state, during which representations of a given motor act are internally rehearsed by the working memory without any overt motor output (Decety, 1996). Jeannerod and Decety (1995) found that mapping of brain activity during MI exhibits an activation pattern similar to that of an executed action. Therefore, we used MI to investigate motor cortex excitability of the dominant untrained side accompanied by movement and motor learning of the trained side, because MI is able to avoid contaminant sensory feedback by actual voluntary movement in the trained limb. The purpose of this study was to examine the effect of intermanual transfer on MI of the untrained side *via* the resulting changes in motor cortex excitability.

## Materials and Methods

### Participants

The participants included 16 healthy individuals [six men and 10 women; mean age ± standard deviation (SD), 21.7 ± 0.3 years] with no history of neurological or psychiatric disease. Handedness was confirmed using the Edinburgh inventory (Oldfield, 1971); 15 participants were right-hand dominant and one participant was left-hand dominant. Written informed consent was obtained from all participants, and the study was approved by the Ethical Committee of the Kanagawa University of Human Services. The experiments were performed in accordance with the Helsinki Declaration. The participants were naïve to both the hypothesis and the stimulation conditions.

### Procedures

**Figure 1:**
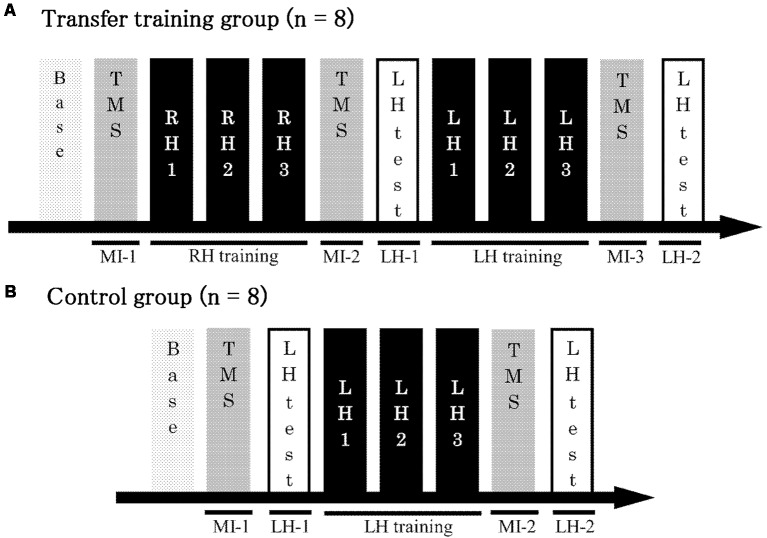
Experimental protocol. **(A)** The transfer training group trained using both the right and left hands. Test trials were conducted to measure motor potentials evoked by transcranial magnetic stimulation (TMS) during MI at three time points in the training process: before right-hand training, after right-hand training but before left-hand training, and after left-hand training. **(B)** The control group received only left-hand training. Test trials were conducted to measure motor potentials evoked by TMS during MI at two time points in the training process: before and after training. Base, Baseline; MI, motor imagery; LH, left hand; RH, right hand; LH-1, LH-2, performance of the left hand.

The participants were randomly assigned to two groups, and each group was evaluated with various tests. The first, the transfer training group, included eight participants (three men and five women; all right-handed; mean age ± SD, 21.6 ± 0.5 years), and the second, the control group, included eight participants (three men and five women; seven right-handed; mean age ± SD, 21.8 ± 0.3 years). The experimental procedure is shown in Figure 1. First, participants in the transfer training group were pre-tested on the right-hand task and on MI for the left hand (MI-1). Next, as a training session, they were asked to perform the conditioned tracking task 30 times using the right hand. After the training session, another test was conducted for MI of the left hand (MI-2), using the same procedure as in the pre-test. Next participants performed a test of the task using their left hand (LH-1). This was the first time that the left hand was actually used. Next, as a training session, participants performed the conditioned tracking task 30 times, using their left hand. Finally, post-tests were conducted for the left-hand task (LH-2) and MI for the left hand (MI-3; Figure 1A). Participants in the control group were trained using only the left hand and evaluated using a pre-test (LH-1, MI-1) and post-test (LH-2, MI-2) of the left hand (Figure 1B).

### Evaluations for Learning Task and Motor Performance

**Figure 2:**
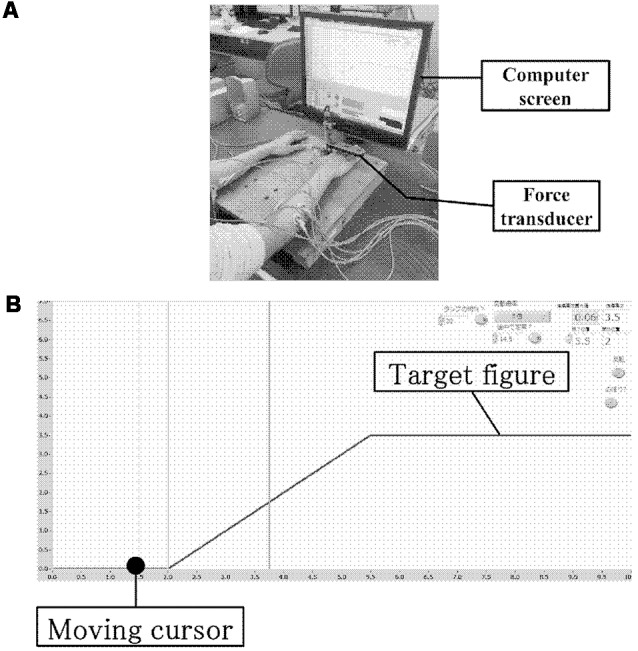
Experimental environment and computer screen. **(A)** Experimental environment. **(B)** Computer screen showing the tracking monitor and tracking figure.

Participants sat comfortably in a chair in front of a table with their right and left forearms positioned horizontally over the table, and the elbow flexed at a 45° angle in the prone position. The right hand was held in a neutral position with slightly extended fingers and attached to a force transducer (SA-250 strain amplifier TEAC, Tokyo, Japan), which the participants could press using the ends of their four metacarpals but without using their thumb (Figure 2A; Sugawara et al., 2016).

All participants were instructed to perform a wrist-extension task while observing a computer screen positioned in front of them. The target figure for tracking comprised a rising ramp slope. The task involved tracking a target with a cursor moving at a steady pace on the computer screen (from left to right). The cursor was moved by the participant as its vertical position on the computer screen corresponded to the wrist extension force measured by the force transducer (Figure 2B). The temporal pattern of target tracking consisted of a silent phase for 2 s, a ramp slope for 3.5 s, and finally a force-holding phase for 4.5 s; thus, the total time was 10 s. As long as an extension force was exerted, the cursor moved towards the top of the screen, and on releasing the grip, it moved towards the bottom of the screen. Prior to the task, the force of maximum voluntary contraction (MVC) for right wrist extension was measured, and the force required during the force-holding phase was set to 30% of the MVC. The same value was also used for the left-hand task. For the silent phase, the force was set to 0% of the MVC. Between tasks, participants rested for 5 s to avoid fatigue. The training task comprised three sessions using the right hand, and each session comprised 10 trials. This training session was measured to provide feedback on the participants’ motor performance during each trial. Online feedback was presented in terms of error values by measuring differences between the target and the actual force output exerted by the participant. In the transfer training group, tests were conducted with the left hand after three training sessions using the right hand, and the effect of MI was evaluated. Following this, three training sessions were conducted using the left hand. For the control group, only the three training sessions using the left hand were conducted.

In the test trials, the target waveform suddenly disappeared 1.5 s after the start of the presentation, and the participant was asked to continue the tracking task by memory without feedback. The motor performance parameter was calculated as the degree of inclination of the actual force output at that time in each trial. The slope was measured in the middle of the rising phase for 2 s as a measure of the differences between the target and actual force and was defined as the average slope of the data points during this 2-s period (calculated as the slope of the least-squares line of best fit). The mean and SD of these average slope values were calculated across 10 trials, and the coefficient of variation (CV) of the slope (CV = SD/mean) was calculated to investigate changes in motor learning. A low slope CV value was considered to indicate stable performance. The timing, presentation of the target figure, feedback information, and order of stimulation were calculated by subtracting the area of the target waveform from the area of the actual output waveform using LabVIEW, ver. 7.1 (National Instruments, Austin, TX, USA).

### Electromyography (EMG) and TMS

During the tracking task, motor cortex excitability was evaluated during MI by measuring the motor evoked potentials (MEPs) produced by TMS. TMS was delivered through a 9-cm diameter figure-of-eight coil connected to a Magstim 200 stimulator (Magstim Co., Whitland, UK), which was placed tangentially to the scalp in the optimal position over the right hemisphere and directed to elicit maximal MEPs in the left extensor carpi radialis (ECR). The coil was placed tangentially to the scalp, with the handle pointing backward, and rotated away from the midline by approximately 45°. The current induced in the brain was therefore directed approximately perpendicular to the line of the central sulcus (Werhahn et al., 1994). Since the MEP recording was performed with the muscles in a resting state, the resting motor threshold (rMT) of the resting muscle was used to define the test intensity. The rMT was defined as the lowest stimulus intensity required to produce MEPs greater than 50 μV in at least 5 of the 10 successive trials during the resting phase of the tested muscle (Rossini et al., 1994). The intensity of the TMS test stimulus was set to 1.2× rMT. This intensity was selected to evoke an obvious response in each muscle at the same time. In the test trials during MI, TMS pulses were delivered 3.7 s after the start, in the middle of the rising phase. Ten trials were performed in each block. Thus, during evaluation of MI, the subject was observing the moving tracking monitor without moving the cursor. To assess changes in MEPs, this test was conducted 10 times for each experiment. Before recording, the difference between the first-person perspective (kinematic imagery) and third-person perspective (visual imagery) was explained to subjects. In order to remind the subject that the first-person image (kinesthetic MI) was not just a third person’s image, we provided the following instruction: “Please imagine the muscle sensation that occurs when performing the muscle contraction.” For comparison, the MEP modification between each condition was recorded at baseline (at rest) 10 times before each experiment. This “baseline” was recorded with the muscle at rest and the subject not thinking about anything. The stimulation conditions were the same as the other condition.

Disposable silver–silver chloride electromyography (EMG) electrodes (1.0 cm in diameter) were placed on the left hand ECR muscles in a belly-tendon montage. The impedance was reduced to below 5 kΩ. EMG signals were amplified using a conventional EMG apparatus (Neuropack; Nihon Kohden, Tokyo, Japan) and bandpass filtered at 3–20 kHz. The signals were then digitized at 4 kHz and fed into a computer for off-line analysis. Background EMG activity in the ECR muscle was calculated by assessing the root mean square (RMS) value over 100 ms before the TMS-evoked activity. These EMG data were moved to a laboratory computer (Labchart AD Instruments Pty Ltd., Bella Vista, NSW, Australia) for off-line analysis. The peak-to-peak amplitudes (mV) of all MEPs for the ECR muscles were calculated offline after completion of the experiment. MEP responses for each condition were expressed as the ratio to the mean value at rest before the experiment (MEP ratio = MEP during MI in test/MEP at rest before the experiment). In addition, the RMS of the background EMG (BEMG) was used to calculate the BEMG ratio (BEMG ratio = BEMG of pre-test during MI/BEMG of post-test during MI).

### Evaluation of Motor Images

The Vividness of Movement Imagery Questionnaire-2 (VMIQ-2) was used to evaluate MI (Roberts et al., 2008). The VMIQ-2 was developed to evaluate the relationship between MI and motor performance and consists of 12 items, in which the subject self-evaluates the vividness of images. Each answer is a number from 1 to 5* (1, maximally vivid; 5, no imagery)*. The subjects were instructed to report “images of physical sensation associated with the motor task,” i.e., on their kinesthetic imagery. The total score was calculated for the 12 items.

### Data Analysis and Statistics

Baseline parameters [age, VMIQ-2 score, performance in pre-test with right hand, and MEP ratio during MI (MI-1)] were compared for the training and control groups using the Mann-Whitney *U* test.

To investigate changes in slope CV before and after training with the left hand, a two-way repeated measures ANOVA was conducted using the training (LH-1 or LH-2) and group (transfer training or control) as factors. *Post hoc* analysis to detect significant differences for the various comparisons was performed using two-tailed *t*-tests with Bonferroni corrections. For the transfer training group, changes in MEPs and BEMG during MI were evaluated using Friedman’s test, and *post hoc* comparisons were performed using the Bonferroni test. For the control group, changes in MEPs and BEMG during MI were evaluated using the Wilcoxon signed-rank test. Changes in MEPs during MI before and after training were evaluated using the Mann-Whitney *U* test. Statistical analyses were performed using IBM SPSS 20 for Windows (IBM Corp., Armonk, NY, USA). For all comparisons, *P*-values less than 0.05 were considered statistically significant.

## Results

### Comparisons of Baseline Data Between the Two Groups

There were no statistically significant differences between the transfer training and control groups regarding age (*P* = 0.442), VMIQ-2 scores (*P* = 0.195), degree of inclination of actual force output by the right hand (*P* = 0.328), or MEP ratio in MI-1 in the pre-training test (Table 1).

**Table 1:** Comparisons of baseline data.

	TTG (*n* = 8)	CG (*n* = 8)	Significant difference
Age	22 (21–22)	22 (21–22)	n.s
VIMQ-2	30 (26–33)	36 (29–36)	n.s
Change of coefficient of variation in slope in right hand	0.44 (0.35–0.57)	0.40 (0.28–0.41)	n.s
MEP ratio of MI-1	2.0 (1.7–2.1)	1.6 (1.4–2.0)	n.s

Median (interquartile range). VMIQ-2, Vividness of Movement Imagery Questionnaire-2; MEP, motor-evoked potential; MI, motor imagery; TTG, transfer training group; CG, control group.

### Changes in Task Performance

**Figure 3:**
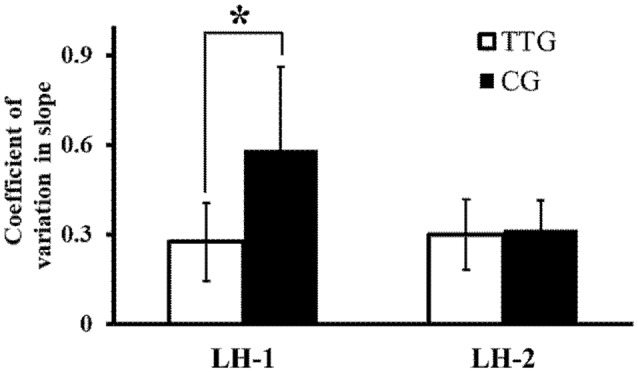
Changes in coefficient of variation (CV) of slope. CV of slope at time points LH-1 and LH-2 for the two groups. The variability in the degree of inclination of the actual force output by the left hand decreased significantly more in the transfer training than in the control group. *p < 0.05.

Figure 3 shows slope CVs for the left hand for the two groups before and after left-hand training. In a two-way repeated measures ANOVA, we found a significant main effect of training (*F*_(1,14)_ = 5.63, *P* = 0.03), a significant main effect of group (*F*_(1,14)_ = 5.50, *P* = 0.03), and a significant interaction between the two factors (*F*_(1,14)_ = 8.10, *P* = 0.01). *Post hoc* analyses revealed that the slope CV before left-hand training (LH-1) was significantly lower in the transfer training than in the control group (*P* = 0.046). *Post hoc* analyses also showed that after left-hand training (LH-2), the difference in slope CV between the two groups was not significant (*P* = 0.067, Figure 3).

### Changes in BEMG Before MEP Testing

**Figure 4:**
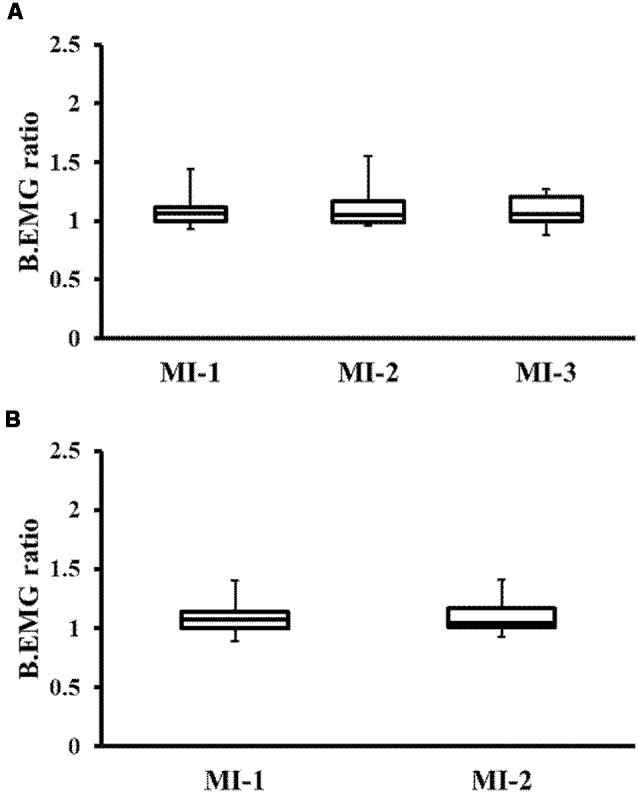
Changes in background electromyography (BEMG) ratio. **(A)** Changes in BEMG of the TTG. **(B)** Changes in BEMG of the CG. There were no significant differences between the groups. TTG, transfer training group; CG, control group.

In the transfer training group, Friedman’s test showed no significant difference in BEMG among the three MI periods (MI-1, MI-2, MI-3; *P* = 0.687; Figure 4A). Similarly, the Wilcoxon signed-rank test showed no significant difference in BEMG in the control group between the two MI periods (MI-1, MI-2; *P* = 0.684; Figure 4B).

### Changes in MEPs During MI in the Transfer Training Group

**Figure 5:**
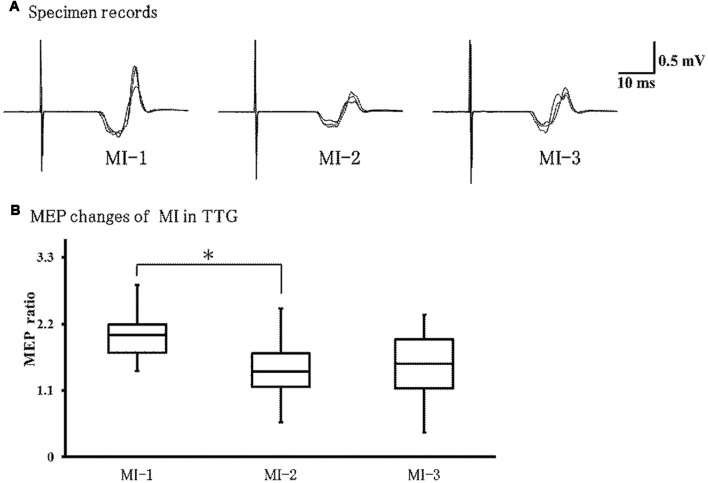
Changes in motor evoked potentials (MEPs) during left-hand MI in the TTG. **(A)** Representative EMG recordings from the extensor carpi radius muscles from a single subject in the transfer training group, across three conditions. **(B)** Quantification of MEP ratio in the three conditions. MEP ratio was significantly lower at MI-2 than at MI-1 (*p* = 0.037). In addition, there were no significant differences between other conditions. *p < 0.05.

Figure 5A shows representative EMG recordings from ECR muscles for a single subject in the transfer training group, across the three conditions. Friedman’s test showed a significant effect of training on MEPs (*P* = 0.01; Figure 5B). *Post hoc* analyses revealed that MEPs were significantly lower in MI-2 than in MI-1 (*P* = 0.037). No other significant differences were observed.

### Changes in MEPs During MI in the Control Group

**Figure 6:**
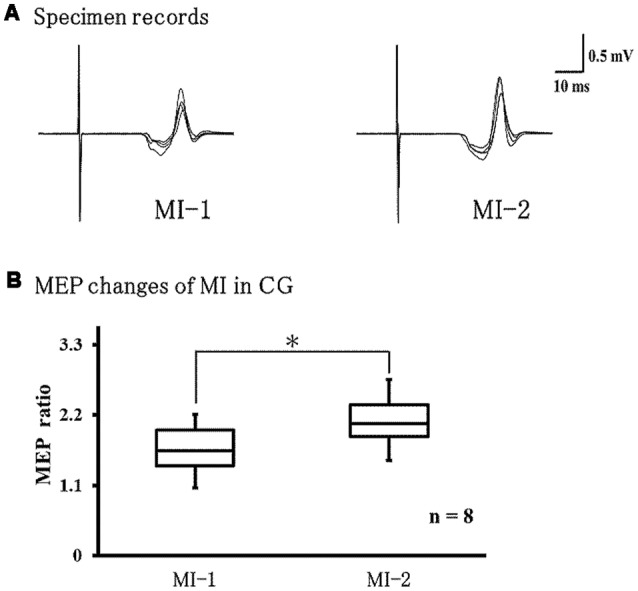
Changes in MEPs during left-hand MI in the control group. **(A)** Representative EMG recordings from the extensor carpi radius muscles from a single subject in the control group, across two conditions. **(B)** Quantification of MEP ratio in the two conditions MEP ratio was significantly larger at MI-2 than at MI-1 (*p* = 0.021). *p < 0.05.

Figure 6A shows representative EMG recordings from the ECR muscles for a single subject in the control group across conditions. We found that MEPs were significantly larger during MI-2 than during MI-1 (*P* = 0.021; Figure 6B).

## Discussion

The results of this study revealed that motor learning of the trained limb changes the motor performance and effects of MI on the untrained side. When tasks were performed for the first time with the left hand, the slope CV was significantly lower in the transfer training than in the control group. As the slope CV indicates the variation in performance in the test trial, the low values observed in the transfer training group suggest a more stable performance. After right-hand training in the transfer training group, MEPs during left-hand MI were significantly decreased, and the decrease was maintained after the actual training of the left hand. In contrast, in the control group, left-hand training in the absence of prior right-hand training significantly increased MEPs during MI. Moreover, when the task performance was initially conducted by the left hand, the slope CV was significantly reduced in the transfer training group. Thus, even though no training was involved, the skilled performance could be conducted by the left hand.

Our results suggest that intermanual transfer might correlate with MI integration of the untrained side and possibly plays an important role in the transition from an initial to a stable performance of the contralateral side.

In this study, MEPs were induced during the subject’s imaged kinesthetic MI. This involves recalling muscle contraction based on a muscle sensory image and was reported to indicate the activity of brain regions similar to those involved in actual muscle contraction (Ruby and Decety, 2001). In the transfer training group, the muscle sensory image evaluation correlated to the actual task execution with the right hand. As a result, it was easy to recall the kinesthetic MI for the training task, thus affecting MI of the non-trained limbs so that MEP changes occurred in the left hand’s MI. In addition, brain excitability during MI for a specific task dynamically changes according to the skill improvement of the subject (Milton et al., 2008). From a neurophysiological perspective, it is considered that intermanual transfer contributes to MI formation of non-practicing limbs and performance stability. As there were different changes in left-hand MI after training between the two groups, training interventions on the ipsilateral and contralateral side may have had different effects on corticospinal excitability.

### Intermanual Transfer Effects on the Untrained Side Performance

Several previous studies have used repeated training to ameliorate the performance of an untrained side of the body. Specifically, they have investigated the effect of intermanual transfer on ballistic motor performance (Carroll et al., 2008; Lee et al., 2010), sequence learning (Dickins et al., 2015; Ossmy and Mukamel, 2016), and eye-hand motor coordination (Fernandez-Ruiz et al., 2006; Veldman et al., 2015). However, performance transfer was not demonstrated in all motor tasks, and the transfer that occurred was task-specific (Lefumat et al., 2015; Romkema et al., 2015).

The tracking task used in this study required fine motor skills for control, using wrist extension force. In contrast, in the training sessions, the task was conducted with visual feedback, and in the test sessions, it was performed without visual feedback. Thus, the test sessions required proprioceptive sensation. Johansson and Westling (1988) demonstrated that for skilled finger motor performance, motor output is more effective and stable after motor learning, which occurs prior to motor output, accompanying proprioceptive sensations. An accumulation of such motor tasks could enable skillful motor learning. Moreover, Gordon et al. (1994) reported that unilateral training of fine motor skills affects the contralateral untrained side, a phenomenon known as intermanual transfer. However, the task of producing a force output by using the index finger and thumb cannot be judged by appearance, as it is used to measure the muscle tension exerted at that time. As a result, tension at one trial is similar to that exerted at the previous trial. Furthermore, this phenomenon is recognized both in the ipsilateral and contralateral limb. In this study, the somatosensory sensation obtained by the right-hand repetition exercise and the fact that subjects performed it with their left hand suggests that stable performance is possible even when doing the task for the first time. In agreement, we showed that repeated unilateral training enhances the stable motor performance of the contralateral side by affecting proprioception and motor memory.

### Unilateral MI Effects on Motor Cortex Excitability and Intermanual Transfer

MEPs produced by TMS are caused by stimulation of motor cortex interneurons, which in turn causes firing of the corticospinal tract cells, leading to a descending volley in the corticospinal tract and, as a result, to α motoneurons firing in the spinal cord, and eventually to muscle contraction (Rothwell, 1997). As the intensity of MI increases, the excitability of the motor cortex is enhanced, which is conducive to achieving motor learning (Facchini et al., 2002; Fourkas et al., 2008). In this study, we found that MEPs during MI significantly decreased by unilateral training in the transfer training group. This suggests that unilateral training decreases MEPs during MI on the contralateral side. Previous studies showing decreased MEPs during motor learning of skilled movements have reported that the underlying mechanism is the surrounding inhibition (Beck and Hallett, 2011; Sugawara et al., 2013). This phenomenon contributes to fine motor skill efficiency and elimination of unnecessary muscle activity *via* inhibition of motor cortex excitability. In our study, MEPs decreased during left-hand MI after learning with the right hand, suggesting that excessive muscle activity is suppressed to yield efficient motor programs. Moreover, in the transfer training group, the decrease in MEPs during MI was maintained after further training of the left hand. Therefore, repeated training of the unilateral side also affects the contralateral untrained side.

### Unilateral MI Effects and Motor Cortex Excitability Without Intermanual Transfer

In the control group, without prior right-hand training, we found a significant increase in MEPs during MI after left-hand training. Perez et al. (2004) demonstrated that repeated unilateral training accompanied by improved task performance increases motor cortex excitability to control agonist muscles. The authors suggested that this increased excitability is reflected in the motor learning process by motor-sensory integration between muscles and the motor cortex. In our study, participants in the control group trained their left hand, so left-hand learning was based on movement execution by the left hand and on sensory feedback in the naïve condition. This suggests that motor execution and sensory feedback generate a dynamic plasticity in the motor cortex, which might increase MEPs during MI after left-hand training.

### Ipsilateral Training Effects After Contralateral Training

We considered that the motor learning process during right-hand training might have affected MEPs during MI post-training of the left hand. Camus et al. (2009) reported that unilateral motor training induces complementary excitability changes in the motor cortex of the right and left hemispheres due to differences between the trained and untrained side. The motor cortex of the trained side showed increased excitability, while motor cortex excitability of the untrained side decreased due to inter-hemispheric disinhibition. This suggests that excitability changes in each neural circuit contribute to skilled performance by the ipsilateral and contralateral sides in the same motor task. It also suggests that the combination of image training and intermanual transfer for recovery of motor functions may be useful in rehabilitation of patients with neurological disorders (e.g., stroke) and for improving muscle weakness in those with musculoskeletal disorders.

## Ethics Statement

Written informed consent was obtained from all participants, and the study was approved by the Ethical Committee of the Kanagawa University of Human Services. The experiments were performed in accordance with the Helsinki Declaration. The participants were naïve to both the hypothesis and the stimulation conditions.

## Author Contributions

RO conducted the whole study and drafted the manuscript. ST made some computer programs to conduct experimental procedures. RO, RI and KS contributed in problem identification. RO and TS collected the experimental data. KS directed the whole experiment, drafted the manuscript, and serves as the corresponding author.

## Conflict of Interest Statement

The authors declare that the research was conducted in the absence of any commercial or financial relationships that could be construed as a potential conflict of interest.
